# Case Report: novel mutations in SMARCA4 cause Coffin-Siris syndrome type 4 with autism spectrum disorder without visual impairment in one patient

**DOI:** 10.3389/fgene.2026.1925563

**Published:** 2026-08-24

**Authors:** Xiuling Chen, John Sieh Dumbuya, Jing Qi

**Affiliations:** Department of Pediatrics, Haikou People’s Hospital, The First Affiliated Hospital of Hainan University, Haikou, China

**Keywords:** autism spectrum disorder, case report, Coffin-Siris syndrome, frameshift mutation, genotype-phenotype correlation, haploinsufficiency, SMARCA4, tumor surveillance

## Abstract

Coffin-Siris syndrome type 4 (CSS4; OMIM 614609) is a rare autosomal dominant disorder caused by variants in SMARCA4, encoding the BRG1 ATPase subunit of the BAF chromatin-remodeling complex. Although classically characterized by intellectual disability, distinctive facial features, and fifth digit hypoplasia, the phenotypic spectrum is broad and includes autism spectrum disorder (ASD) without typical somatic features. We report a 3-year-old Chinese girl with global developmental delay, ASD features, characteristic facial features, and a documented normal ophthalmologic examination in whom whole-exome sequencing identified a novel heterozygous *de novo* frameshift duplication, c.4767dup (p.Ser1590Ilefs*39), in SMARCA4 (NM_003072), classified as Pathogenic (PVS1+PM2_Supporting + PM6). Notably, she lacked fifth digit hypoplasia and had no structural ocular anomalies on detailed ophthalmologic evaluation. A systematic review of the literature identified 39 genetically confirmed SMARCA4-related CSS4 cases; combined with our patient, 40 cases were analyzed. Intellectual disability was universal (100%), ASD manifestations occurred in 42.5%, and fifth digit hypoplasia was present in only 55.0%. Truncating variants (37.5% of the cohort) showed descriptive trends toward higher prevalence of fifth digit hypoplasia and ocular abnormalities than missense variants, though these differences did not reach statistical significance. Our proband, despite carrying a truncating variant, presented without classic digital anomalies or ocular involvement, underscoring that even loss-of-function alleles can produce atypical CSS4 phenotypes. For children with developmental delay, ASD, and distinctive facial features—regardless of the presence of classic digital anomalies—genetic testing for SWI/SNF complex genes should be considered. Given current limited evidence, carriers of truncating variants should be counseled regarding potential tumor predisposition, and individualized surveillance strategies may be considered pending further data.

## Introduction

Coffin-Siris syndrome (CSS) is a rare autosomal dominant disorder caused by pathogenic variants in genes encoding subunits of the BRM-associated factor (BAF) chromatin-remodeling complex (Vasko et al., 2021). The SMARCA4 gene, located at 19p13.2, encodes the core ATPase subunit BRG1 and plays a critical role in neural development and cardiac morphogenesis (Kos et al., 2014). CSS type 4 (CSS4; OMIM 614609), resulting from SMARCA4 variants, accounts for approximately 11%–15% of all CSS cases (Sekiguchi et al., 2019; Kosho et al., 2014).

Autism spectrum disorder (ASD) is a neurodevelopmental condition characterized by impaired social communication and restricted, repetitive behaviors. Recent large-scale sequencing studies have implicated SWI/SNF complex genes, including SMARCA4 and ARID1B, in ASD susceptibility (Satterstrom et al., 2020; De Rubeis et al., 2014). The clinical phenotype of SMARCA4-related CSS4 spans a broad spectrum, ranging from classic CSS to non-syndromic ASD (Li et al., 2020; Qian et al., 2022). Additionally, ocular developmental abnormalities (e.g., microphthalmia and optic nerve hypoplasia) are increasingly recognized as extended phenotypes (Chesneau et al., 2026; Errichiello et al., 2017). While previous reports have described truncating SMARCA4 variants without documented ocular anomalies, the absence of comprehensively evaluated normal eye examinations limits phenotypic characterization. Systematic reports on CSS4 with ASD from China remain limited. Here, we describe a Chinese child with CSS4 and ASD caused by a novel *de novo* SMARCA4 truncating variant, and analyze genotype-phenotype associations using data from 39 patients reported in the literature.

## Case description

The patient, a 3-year-old girl, presented to the Pediatric Developmental and Behavioral Specialty Clinic of Haikou People’s Hospital in April 2025 because of language delay lasting more than 1 year. She was the first child of non-consanguineous parents (G1P1), born at full term *via* vaginal delivery with a birth weight of 3.2 kg. Developmental milestones included head control at 3 months, independent sitting at 6 months, crawling at 9 months, and independent walking at 13 months; however, she had not acquired meaningful speech. Physical examination revealed a height of 92.5 cm (<3rd centile), weight of 14.4 kg (<3rd centile), and head circumference of 46 cm (Table 1). Characteristic facial features included thick eyebrows, long eyelashes, and a flat nasal bridge. No hypoplasia of the fifth fingers or toes was observed. There was no specific family history of neurodevelopmental disorders or congenital anomalies.

**TABLE 1:** Clinical characteristics and genetic findings of the proband.

Category	Finding
Demographics	
Sex	Female
Age at presentation	3 years
Birth history	G1P1, full-term vaginal delivery, birth weight 3.2 kg
Family history	Non-consanguineous parents; no specific family history
Growth parameters	
Height	92.5 cm (<3rd centile)
Weight	14.4 kg (<3rd centile)
Head circumference	46 cm
Dysmorphic features	Thick eyebrows, long eyelashes, flat nasal bridge; no fifth digit hypoplasia
Developmental milestones	
Head control	3 months
Independent sitting	6 months
Crawling	9 months
Independent walking	13 months
Meaningful speech	Absent at 36 months
Neuropsychological assessment	
Gesell developmental schedule (total DQ)	52.5
Gross motor (equivalent age)	24 months
Fine motor (equivalent age)	15 months
Adaptive behavior (equivalent age)	25.5 months
Language (equivalent age)	9 months
Personal–social behavior (equivalent age)	21 months
Autism behavior checklist (ABC)	94
Social maturity scale (S-M scale)	8
Behavioral phenotype	No response to name, brief eye contact, does not follow instructions, infrequent peer play, solitary play, stereotyped behaviors (lining up toys)
Electroencephalography	Generalized delta activity with sharp slow-wave discharges
Cranial MRI	Thinning of the corpus callosum
Audiometry (ABR)	Left ear: 25 dBnHL; right ear: 30 dBnHL (DPOAE present bilaterally)
Ophthalmological examination	No microphthalmia, optic nerve hypoplasia, retinal dystrophy, or significant refractive error
Echocardiography	Normal
Genetic findings	
Gene	SMARCA4
Transcript	(NM_003072)
Variant (cDNA)	c.4767 dup
Variant (protein)	p.S1590Ifs*39 (p.Ser1590Ilefs*39)
Location	Exon 33 (C-terminal region)
Variant type	Heterozygous frameshift duplication, *de novo*
Protein domain	Helicase ATP-binding domain (catalytic core region)
ACMG classification	Pathogenic (PVS1 + PM2_Supporting + PM6)

Abbreviations: DQ, developmental quotient; ABR, auditory brainstem response; DPOAE, distortion product otoacoustic emissions; ACMG, american college of medical genetics and genomics.

At presentation, she exhibited limited speech, did not respond to her name, showed brief eye contact, failed to follow instructions, engaged infrequently in peer play, preferred solitary play, and exhibited stereotyped behaviors (lining up toys, picking at small holes, and playing with sand). The Gesell Developmental Schedule (GDS) yielded a total developmental quotient of 52.5, with subdomain equivalent ages as follows: gross motor 24 months, fine motor 15 months, adaptive behavior 25.5 months, language 9 months, and personal-social behavior 21 months. The Autism Behavior Checklist (ABC) score was 94, and the Social Maturity Scale (S-M Scale) score was 8. Standardized HPO phenotype terms included autism spectrum disorder (HP: 0000717) and delayed speech and language development (HP: 0000750).

Auxiliary investigations showed EEG generalized delta activity with sharp slow-wave discharges in the absence of clinical seizures. Cranial MRI demonstrated thinning of the corpus callosum. Auditory brainstem response testing revealed air-conduction thresholds of 25 dBnHL in the left ear and 30 dBnHL in the right ear; distortion product otoacoustic emissions were present at most frequencies bilaterally. A comprehensive ophthalmologic examination was performed, including visual acuity assessment, slit-lamp biomicroscopy, dilated fundoscopy, and evaluation for strabismus and nystagmus. No structural abnormalities (e.g., microphthalmia, microcornea, optic nerve hypoplasia) or refractive errors were identified. Binocular visual acuity testing showed no significant refractive error. Echocardiography was normal. A summary of the auxiliary investigations is provided in Supplementary Figure S1.

## Diagnostic assessment

Whole-exome sequencing was performed at 18 months of age (October 2023) using the IDT xGen Exome Research Panel v2.0 for capture and the Illumina NovaSeq 6000 platform for sequencing. Target region coverage was 99.60%, with >20× coverage in 98.30% of the target region. Peripheral blood samples were collected from the proband and her parents. Genomic DNA was extracted and sequenced. Suspected variants were validated by Sanger sequencing using an ABI 3730 sequencer.

**FIGURE 1:**
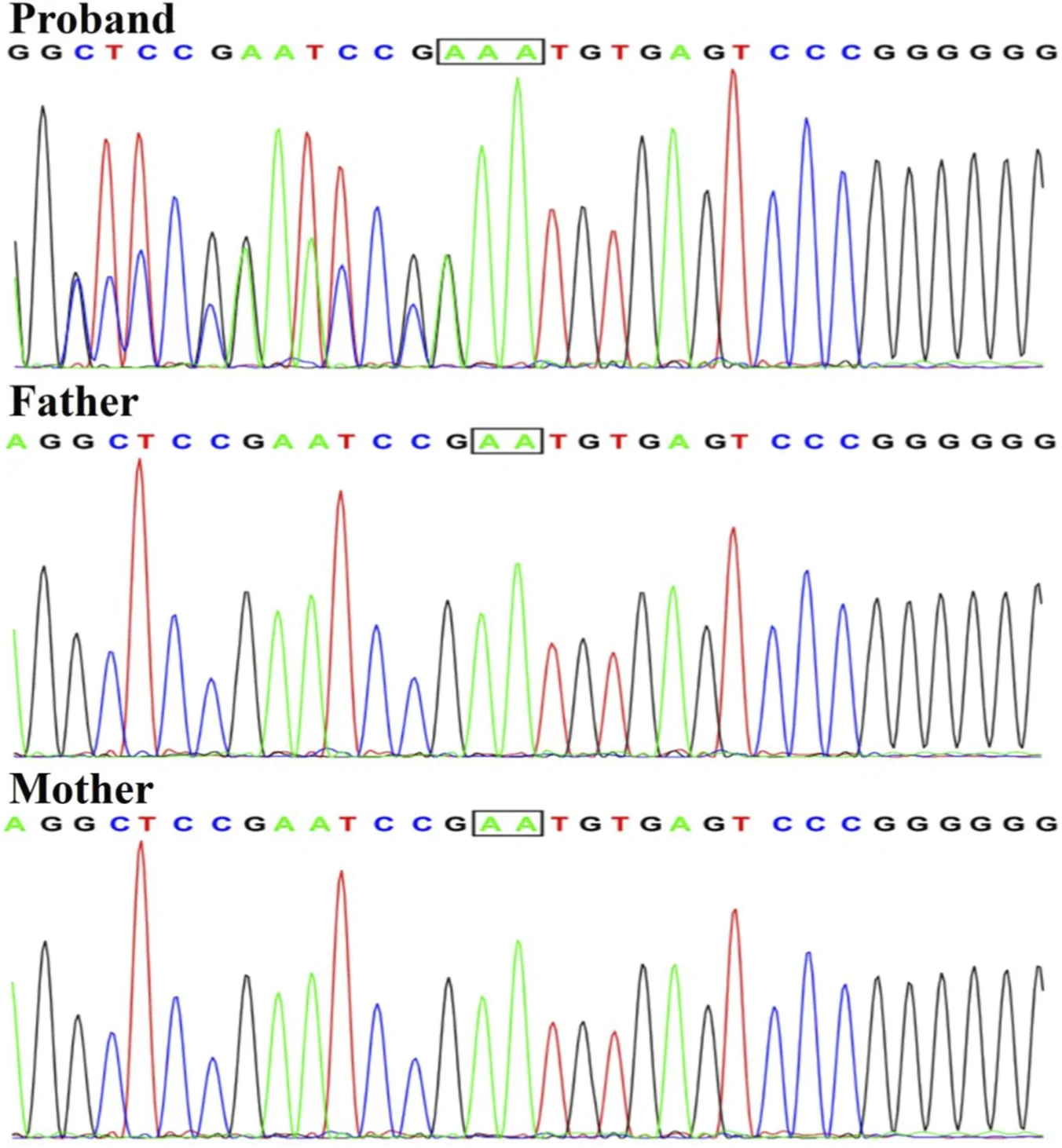
Schematic representation of the SMARCA4 (BRG1) protein and the location of the novel c.4767dup (p.Ser1590Ilefs*39) frameshift duplication variant identified in this case. The linear protein map depicts the major functional domains: QLQ (glutamine-leucine-glutamine-rich), HSA (helicase-SANT-associated), Pro-rich (proline-rich), BRK (BRG1/KIAA0154-related), Helicase ATP-binding, Helicase C-terminal, Bromo (bromodomain), and CTD (C-terminal domain). The dashed bracket indicates the catalytic core region (amino acids 766–1,246). The red arrow marks the frameshift duplication variant located within the helicase ATP-binding domain. The scale bar represents 200 amino acids. N, amino terminus; C, carboxyl terminus.

Whole-exome sequencing identified a heterozygous frameshift duplication, c.4767dup (p.Ser1590Ilefs*39), in SMARCA4 (NM_003072). The variant is located in exon 33 and is predicted to introduce a premature termination codon 39 residues downstream. While premature termination codons in terminal exons often escape nonsense-mediated mRNA decay (NMD), this frameshift is predicted to produce a truncated BRG1 protein lacking critical C-terminal domains (including the helicase C-terminal and bromodomain regions), rendering it non-functional regardless of NMD status. Sanger sequencing confirmed the variant in the proband and its absence in both parents, establishing a *de novo* origin (Figure 1). According to ACMG criteria, the variant was classified as Pathogenic (PVS1: null variant in a gene where loss-of-function is a known disease mechanism; PM2_Supporting: absent from population databases at a threshold of <0.0005; PM6: detected *de novo* in one or more probands with the phenotype) (Ric et al., 2015). The clinical timeline is shown in Supplementary Figure S2.

## Literature review and results

A systematic search was performed in PubMed, Web of Science, and the China National Knowledge Infrastructure (CNKI) using the search strings: (“SMARCA4” OR “BRG1”) AND (“Coffin-Siris syndrome” OR “CSS4” OR “autism spectrum disorder” OR “intellectual disability”) AND (“eye anomaly” OR “ocular” OR “microphthalmia” OR “optic nerve hypoplasia”). No language restrictions were applied. We included case reports and series of SMARCA4-related CSS4 confirmed by genetic testing with detailed phenotypic data, and excluded studies reporting variants of uncertain significance, overlapping SWI/SNF complex disorders without SMARCA4confirmation, or duplicate patient reports. Two reviewers (JSD and JQ) independently screened titles/abstracts and performed full-text reviews; discrepancies were resolved by consensus with XC. Duplicate removal was performed using Zotero. This review was conducted in accordance with PRISMA 2020 guidelines for systematic reviews; a PRISMA-style flow diagram is provided in Supplementary Figure S5. Ultimately, 39 patients from the published literature (Kos et al., 2014; Sekiguchi et al., 2019; Li et al., 2020; Qian et al., 2022; Chesneau et al., 2026; Errichiello et al., 2017; Santen et al., 2013; Schmetz et al., 2024) were included; combined with our case, 40 cases were analyzed.

The 39 published cases plus our patient yielded 40 individuals with genetically confirmed SMARCA4-related CSS4. Twenty-three were male (57.5%) and 17 female (42.5%). Diagnostic age ranged from 6 to 40 years (median 8 years). Missense variants accounted for 62.5% (25/40), predominantly clustered within the catalytic core region (residues 766–1,246); truncating variants (nonsense, frameshift, or deletion) accounted for 37.5% (15/40). Our proband belongs to the truncating variant group.

Intellectual disability was present in all cases (100%, 40/40), with moderate-to-severe impairment in 67.5% (27/40); language delay occurred in 92.5% (37/40); and motor delay in 75.0% (30/40). Behavioral characteristics were reported in 74.4%, including ASD manifestations in 42.5% (17/40). Characteristic facial features were noted in 85.0% (34/40); fifth digit hypoplasia occurred in only 45.0% (18/40) and showed a descriptive trend toward higher prevalence in the truncating variant group than in the missense variant group (60.0% [9/15] vs. 36.0% [9/25]; p = 0.17, Fisher’s exact test), though this difference was not statistically tested due to limited sample size. Ocular developmental anomalies were present in 17.5% (7/40), with a descriptive trend toward higher incidence in the truncating variant group (26.7% [4/15] vs. 12.0% [3/25]; p = 0.36, Fisher’s exact test). Cardiac malformations occurred in 37.5% (15/40); brain structural abnormalities in 35.0% (14/40); and epilepsy in 22.5% (9/40). Among the 15 patients with truncating variants, one female (6.7%) developed ovarian hypercalcemic small cell carcinoma (SCCOHT) at age 16 (Errichiello et al., 2017).

A summary of the phenotypic features is provided in Supplementary Table S1 and illustrated in Supplementary Figure S3. The comparison between missense and truncating variants is presented in Supplementary Table S2; Supplementary Figure S4. The literature search process is detailed in Supplementary Figure S5. Detailed characteristics of the published cases are listed in Supplementary Table S3.

## Discussion

The c.4767dup (p.Ser1590Ilefs*39) variant identified in this case is located in exon 33 of SMARCA4, the final exon of the major transcript, and introduces a premature termination codon 39 residues downstream. As a null allele, this frameshift is predicted to undergo nonsense-mediated mRNA decay, resulting in haploinsufficiency and complete loss of BRG1 function. Because the variant resides in the last exon, it may escape nonsense-mediated mRNA decay and produce a truncated protein that retains partial function, rather than resulting in complete haploinsufficiency. This possibility is important to consider, as the phenotypic absence of both fifth digit hypoplasia and ocular abnormalities in our patient might be explained by residual protein activity. However, without functional studies, the actual molecular consequence remains uncertain. Regardless, the variant is a null allele that disrupts the helicase ATP-binding domain, and it likely impairs BRG1 function through either haploinsufficiency or dominant-negative effects. This mechanism contrasts with missense variants in the catalytic core, which may produce dominant-negative effects through disrupted ATPase activity (Li et al., 2020; Qian et al., 2022). Whether the observed phenotype results from haploinsufficiency, dominant-negative effects, or a combination thereof remains to be determined experimentally. Our patient presented with ASD as the predominant neurobehavioral manifestation and lacked the classic fifth digit hypoplasia often regarded as a hallmark of CSS, underscoring the substantial clinical heterogeneity of SMARCA4-related disease—even among carriers of truncating alleles.

The literature review confirms that the phenotypic spectrum of SMARCA4-related CSS4 is continuous: at one extreme lies classic CSS (intellectual disability, digital anomalies, distinctive facial features), and at the other, non-syndromic ASD (Qian et al., 2022). Fifth digit hypoplasia was observed in only 55.0% of cases overall, with a descriptive trend toward lower prevalence among patients with missense variants. Consequently, the absence of digital anomalies cannot be used to exclude a diagnosis of CSS4—a conclusion consistent with the findings of Li et al. (2020). Our proband, despite carrying a truncating variant typically associated with more classic CSS features, did not exhibit fifth digit hypoplasia, further reinforcing this point. ASD manifestations were present in 42.5% of this cohort, a rate substantially higher than that observed in the general population with intellectual disability. Animal studies have demonstrated that SMARCA4 deletion impairs cortical neuronal migration and disrupts GABAergic interneuron differentiation (Paulsen et al., 2022), providing a plausible anatomical substrate for ASD pathogenesis.

Ocular abnormalities, an important extended phenotype of CSS4, occurred in 17.5% of the cohort. Chesneau et al. (2026) systematically described the spectrum of ocular developmental anomalies associated with SMARCA4 loss-of-function variants. Furthermore, our descriptive analysis showed that ocular phenotypes appear more common among truncating variants (26.7% vs. 12.0% in missense variants). However, our proband—who carries a *de novo* truncating frameshift variant—exhibited a completely normal ophthalmologic examination. This case demonstrates that truncating SMARCA4 variants do not universally produce ocular phenotypes and that the expressivity of ocular features is variable even among null allele carriers. This expands the phenotypic spectrum of SMARCA4-related disease and underscores the importance of formal ophthalmologic evaluation in all patients, regardless of variant type. Notably, Li et al. (2020) described two individuals with novel nonsense variants who had milder phenotypes with no organ-system involvement; importantly, no ophthalmologic abnormalities were reported for these patients, although the absence of a comprehensive eye examination was not explicitly documented. Our case therefore contributes a formally evaluated normal ocular examination to the literature on SMARCA4 truncating variants.

Thinning of the corpus callosum was identified in our proband on cranial MRI. Brain structural abnormalities were present in 35.0% of our cohort, with thinning of the corpus callosum being among the most frequently reported findings—consistent with prior reports by Santen et al. (2013); Schmetz et al. (2024). Our patient’s isolated callosal thinning aligns with this pattern, though less severe than ventriculomegaly or cortical malformations described in some cases. However, the functional significance of isolated corpus callosum thinning in the absence of other structural anomalies remains unclear, and longitudinal neuroimaging studies are needed to assess whether this finding correlates with specific neurodevelopmental outcomes. Additionally, epileptiform EEG abnormalities were present in 22.5% of patients, with only a subset manifesting clinical seizures. The presence of generalized slowing with sharp slow-wave discharges suggests a diffuse cortical dysfunction, potentially related to disrupted GABAergic interneuron differentiation—a mechanism implicated in SMARCA4-related neurodevelopmental pathology. While our patient has not experienced clinical seizures, the EEG abnormalities warrant continued monitoring, as electrographic seizures may emerge with age or during periods of physiological stress, in accordance with recommendations for baseline EEG screening in CSS4 even in asymptomatic individuals (Vasko et al., 2021).

Tumor susceptibility is an important consideration for carriers of SMARCA4 truncating variants. Germline loss-of-function variants have been associated with rhabdomyosarcoma predisposition syndrome type 2 (RTPS2) and small cell carcinoma of the ovary hypercalcemic type (SCCOHT) (Errichiello et al., 2017; Del Baldo et al., 2021), although the absolute risk remains poorly quantified. In our reviewed cohort of 40 cases, only one female with a truncating variant developed SCCOHT at age 16 (Errichiello et al., 2017). Currently, no standardized surveillance guidelines exist for SMARCA4 truncating variant carriers. Given the limited evidence, we suggest that clinicians discuss potential tumor risks with families and consider individualized surveillance strategies—such as periodic clinical examination and awareness of relevant symptoms—rather than mandating formal surveillance protocols. Future multicenter collaborations are needed to establish evidence-based screening recommendations. Because our patient carries a truncating null allele, she is at elevated lifetime risk for SMARCA4-deficient tumors. Long-term, structured surveillance—including abdominal and pelvic imaging, clinical examination, and tumor-marker monitoring—should be instituted and maintained through transition to adult care. This represents a major shift in management compared with missense carriers, for whom tumor risk is generally considered lower.

In summary, for children presenting with developmental delay, ASD, and distinctive facial features, genetic testing for SWI/SNF complex genes, including SMARCA4, should be considered regardless of whether classic digital anomalies are present. Accurate molecular diagnosis, particularly the distinction between truncating and missense variants, facilitates prognostic assessment, individualized early intervention, and family genetic counseling. Carriers of truncating variants, such as our proband, should receive balanced counseling regarding potential tumor risk, with surveillance strategies tailored to individual circumstances pending further evidence.

## Conclusion

This case highlights that SMARCA4 truncating variants can cause CSS4 with ASD manifestations even in the absence of classic digital anomalies or ocular involvement. The novel *de novo* frameshift c.4767dup (p.Ser1590Ilefs*39) expands the genotype-phenotype spectrum of SMARCA4-related disease. Clinicians should maintain a high index of suspicion for SWI/SNF complex disorders in children with developmental delay, ASD, and distinctive facial features, irrespective of fifth digit status. Molecular confirmation enables tailored management, including balanced tumor risk counseling and consideration of individualized surveillance for truncating variant carriers.

## Data Availability

The raw whole-exome sequencing data are not publicly available due to privacy and ethical restrictions. De-identified clinical data and the literature review dataset are available from the corresponding author upon reasonable request.
