# Supplementary material for: Case Report: novel mutations in SMARCA4 cause Coffin-Siris syndrome type 4 with autism spectrum disorder without visual impairment in one patient
Source: Front Genet. 2026 Aug 24;17:1925563. doi: 10.3389/fgene.2026.1925563 (PMC13546743; doi:10.3389/fgene.2026.1925563)
Supplement: Supplementary file 1 [file DataSheet1.docx]

**Supplementary Materials**

**Supplementary Figure S1. Summary of auxiliary investigations in the proband.** (A) Developmental profile assessed by the Gesell Developmental Schedule, showing equivalent ages across five domains relative to the chronological age of 36 months (red dashed line). Language showed the most severe delay (9 months). (B) Auditory brainstem response thresholds, showing mild elevation in the right ear (30 dBnHL) compared with the left ear (25 dBnHL); both are near the upper limit of normal for age. Distortion product otoacoustic emissions were present bilaterally. (C) Electroencephalography findings demonstrating generalized slowing with delta activity predominance, sharp slow-wave discharges, and epileptiform abnormalities in the absence of clinical seizures. (D) Cranial MRI findings showing isolated thinning of the corpus callosum with otherwise normal intracranial structures, including normal ventricular size, no focal parenchymal lesions, normal posterior fossa, and no midline structural defects.

**Supplementary Figure S2. Clinical timeline and developmental milestones of the proband.** Timeline depicting the proband's developmental trajectory from birth to presentation at 36 months. Major motor milestones (head control, sitting, crawling, walking) were attained at relatively typical ages, whereas language development was markedly delayed (equivalent to 9 months at chronological age 36 months). The red dashed line indicates the age at genetic diagnosis. Behavioral features including absent response to name, limited eye contact, and stereotyped behaviors (lining up toys) were prominent at presentation.

**Supplementary Figure S3.** Frequency of clinical and developmental features in 40 patients with SMARCA4-related Coffin–Siris syndrome type 4. Horizontal bar chart showing the prevalence of major phenotypic features compiled from 39 published cases and the proband described in this report.

**Supplementary Figure S4.** Comparison of phenotypic features between missense and truncating SMARCA4 variants in 40 patients with Coffin–Siris syndrome type 4. Grouped bar chart comparing the prevalence of key features between patients with missense variants (n=25) and truncating variants (n=15).

**Supplementary Figure S5.** PRISMA-style flow diagram of the literature search and case selection for the systematic review of SMARCA4-related Coffin–Siris syndrome type 4.

**Supplementary Table S1.** Summary of phenotypic features in 40 patients with SMARCA4-related Coffin–Siris syndrome type 4

| **Feature** | **n/N** | **Percentage** |
| --- | --- | --- |
| **Sex distribution** |  |  |
| Male | 23/40 | 57.5% |
| Female | 17/40 | 42.5% |
| **Neurodevelopment** |  |  |
| Intellectual disability (any severity) | 40/40 | 100.0% |
| Moderate-to-severe intellectual disability | 27/40 | 67.5% |
| Language delay | 37/40 | 92.5% |
| Motor delay | 30/40 | 75.0% |
| **Behavioral disorders** |  |  |
| Any behavioral abnormality | 29/39* | 74.4% |
| ASD manifestations | 17/40 | 42.5% |
| **Somatic features** |  |  |
| Characteristic facial features | 34/40 | 85.0% |
| Fifth digit hypoplasia | 18/40 | 45.0% |
| **Ocular abnormalities** |  |  |
| Any ocular developmental anomaly | 7/40 | 17.5% |
| Microphthalmia / microcornea | 5/40 | 12.5% |
| Optic nerve hypoplasia | 4/40 | 10.0% |
| Retinal dystrophy | 1/40 | 2.5% |
| **Other systemic abnormalities** |  |  |
| Cardiac malformations | 15/40 | 37.5% |
| Brain structural abnormalities | 14/40 | 35.0% |
| Epilepsy | 9/40 | 22.5% |
| Tumor risk (truncating variants only, n = 14) |  |  |
| Ovarian small cell carcinoma (SCCOHT) | 1/14 | 7.1% |

*Behavioral abnormalities were reported in 29 of 39 literature cases.

**Supplementary Table S2.** Genotype–phenotype correlation by variant type in 40 SMARCA4-related CSS4 cases

| **Phenotypic Feature** | **Missense variants (n = 25)** | **Truncating variants (n = 15)** | **p-value (Fisher’s exact test)** |
| --- | --- | --- | --- |
| Fifth digit hypoplasia | 36.0% (9/25) | 60.0% (9/15) | 0.17 |
| Ocular abnormalities | 12.0% (3/25) | 26.7% (4/15) | 0.23 |
| ASD manifestations | 40.0% (10/25) | 46.7% (7/15) | 0.79 |
| Cardiac malformations | 36.0% (9/25) | 40.0% (6/15) | **>0.05** |
| Brain structural abnormalities | 32.0% (8/25) | 40.0% (6/15) | **>0.05** |
| Epilepsy | 20.0% (5/25) | 26.7% (4/15) | **>0.05** |

*Exact counts for the fifth digit and ocular abnormalities were extractable from published case-level data; remaining categories represent cohort-level estimates stratified by variant type.

**Supplementary Table S3.** Characteristics of published SMARCA4-related CSS4 cases included in the literature review

| **Reference** | **Year** | **Cases (n)** | **Sex (M/F)** | **Variant type(s) reported** | **Key phenotypes** |
| --- | --- | --- | --- | --- | --- |
| Kosho & Okamoto [2] | 2014 | 4 | 2/2 | Missense, truncating | ID, facial features, fifth digit hypoplasia, cardiac defects, hypotonia |
| Santen et al. [9] | 2013 | 3 | 2/1 | Missense, truncating | ID, hypotonia, facial features, fifth digit hypoplasia, feeding difficulties |
| Sekiguchi et al. [3] | 2019 | 5 | 3/2 | Mixed | ID, language delay, variable fifth digit hypoplasia, cardiac anomalies in subset |
| Li et al. [5] | 2020 | 6 | 4/2 | Nonsense, missense | ID, ASD features, variable fifth digit hypoplasia, milder phenotype with missense variants |
| Qian et al. [6] | 2022 | 8 | 5/3 | Missense, splice‑site | Prominent autistic features, language delay, mild dysmorphism, fewer classic CSS features |
| Chesneau et al. [7] | 2025 | 5 | 3/2 | Loss‑of‑function | Ocular anomalies (microphthalmia, microcornea, optic nerve hypoplasia), ID, facial features |
| Errichiello et al. [8] | 2017 | 1 | 0/1 | Truncating (frameshift) | CSS, microphthalmia, SCCOHT at age 16 years |
| Schmetz et al. [10] | 2024 | 6 | 3/3 | Mixed | Adult phenotype delineation; ID, behavioral issues, scoliosis, renal anomalies in subset |
| Wang et al. [11] | 2024 | 1 | 1/0 | Missense | Neonatal presentation, hypotonia, feeding difficulties, facial features, fifth digit hypoplasia |
| Total literature cases |  | 39 | 23/16 | Missense 24 (61.5%), Truncating 14 (35.9%) | – |
| Plus proband (this report) | – | 1 | 0/1 | Truncating (frameshift) | ASD, developmental delay, facial features, NO fifth digit hypoplasia, NO ocular anomalies |
| Overall (literature + proband) |  | 40 | 23/17 | Missense 25 (62.5%), Truncating 15 (37.5%) | – |

**Abbreviations:** ID, intellectual disability; ASD, autism spectrum disorder; SCCOHT, small cell carcinoma of the ovary hypercalcemic type; CSS, Coffin–Siris syndrome.
